# The efficiency and regimen choice of adjuvant chemotherapy in biliary tract cancer: A STROBE-compliant retrospective cohort study

**DOI:** 10.1097/MD.0000000000013570

**Published:** 2018-12-14

**Authors:** Luxi Yin, Qi Xu, Jingjing Li, Qing Wei, Jieer Ying

**Affiliations:** a Department of Medical Oncology, Zhejiang Cancer Hospital, Hangzhou, Zhejiang; b State Key Laboratory of Molecular Oncology and Department of Etiology & Carcinogenesis, Cancer Institute and Hospital, Chinese Academy of Medical Sciences and Peking Union Medical College, Beijing, China.

**Keywords:** adjuvant chemotherapy, biliary tract cancer, combination chemotherapy, oral agent

## Abstract

Biliary tract cancer (BTC) patients have poor prognosis even following radical resection. To improve the current status, more evidence is required clarifying the role of adjuvant chemotherapy. This study aim is to evaluate the efficacy of adjuvant chemotherapy and discuss the regimen choices.

We retrospectively analyzed the clinical data of 80 patients who underwent curative-intent R0 resection from 2008 to 2016. Among them, 40 patients had received adjuvant chemotherapy, and the others in the observation group were 1:1 matched by clinical characteristics including gender, age, tumor stage, and ECOG performance status score. Kaplan–Meier analysis was performed to compare DFS and OS. Potential confounding factors were adjusted by Multivariate analysis.

In the entire patient cohort, the mean disease-free survival (DFS) time of BTC patients with adjuvant chemotherapy and observation was 18.63 ± 3.63 months versus 10.36 ± 1.67 months, respectively (*P* = .029). There was no significant difference observed in overall survival (OS) time (33.72 ± 5.02 vs 21.05 ± 4.12 months, *P* = .114). On multivariate analysis, adjuvant chemotherapy and N factor were found to be significant factors for DFS, and sex, age, T factor were found to be significant factors for OS. Besides, subgroup analysis indicated that combination chemotherapy prolonged DFS time of BTC patients than single-agent to some extent, and oral agents showed efficacy to improve OS.

This retrospective study demonstrates that adjuvant chemotherapy contributes to DFS, but is unsatisfactory for improving OS. Combination chemotherapy contained oral agents provides a possibility of therapeutic strategy for improving surgical outcomes of BTC patients.

## Abstract 2

Supplemental Digital Content is available in the text

## 1 Introduction

Biliary tract cancers (BTC), are comprised of the following cancers, gallbladder carcinomas (GBC), intrahepatic cholangiocarcinomas (ICC), hilar cholangiocarcinomas (HC), and extrahepatic cholangiocarcinomas (ECC). More than 90% of BTCs are adenocarcinomas and the rest mainly originate from squamous cell.^[1]^ Despite the diversity of these tumors, they all have poor prognosis. Surgery is considered as the only potentially curative treatment option.^[2,3]^ However, only 10% of patients at initial presentation are considered surgical candidates.^[4]^ After radical resection, the 5-year overall survival (OS) rates are 5% to 10% for patients with gallbladder cancer and 10% to 40% for patients with cholangiocarcinoma.^[5]^ In metastatic cases, median survival is no longer than 8 to 12 months even when patients underwent systemic therapies.^[6]^

Due to high rates of disease recurrence and poor survival rates following surgical resection, postoperative treatments including adjuvant radiotherapy, chemotherapy, and concurrent chemo-radiotherapy been tried to improve patients’ survival. There was not sufficient evidence supporting the efficiency of adjuvant therapy. An evaluation of the National Cancer Database (NCDB) carried out by Kalyan C. Mantripragada et al indicated that patients with T2–3 or node-positive, non-metastatic gallbladder cancer, did not achieve a better 3-year OS after adjuvant therapy. Moreover, chemo-radiation may merely provide a short-term benefit in locally advanced tumors.^[7]^ Se-Il Go et al collected clinical data from 84 and 279 patients who were treated with adjuvant therapy and followed up with surveillance only, respectively. Their results revealed that fluoropyrimidine-based adjuvant therapy did not improve the 5-year RFS (recurrence-free survival) and OS rates.^[8]^ However, an analysis of the National Cancer Data Base from 2005 to 2013 including 5029 patients diagnosed with T1-3N0-1 GBC and treated with surgical resection demonstrated a strong association between adjuvant therapy and improved 3-year OS.^[9]^ PRODIGE 12-ACCORD 18 is a multicenter prospective, randomized phase III trial examining the role of adjuvant chemotherapy with gemcitabine-oxaliplatin combination (GEMOX) in patients following R0 or R1 resection of a BTC. Patients were tolerant to chemotherapy but did not show clinical benefit. Another multicenter prospective, randomized phase III trial BILCAP revealed the efficiency of adjuvant chemotherapy with capecitabine, the median DFS time in capecitabine group and observation is 25.9 versus 17.6 months respectively (*P* = .011), and the median OS time is 52.7 versus 36.1 months respectively (*P* = .028). National Comprehensive Cancer Network (NCCN) Guidelines Version 1.2018 recommends 4 choices of treatment for R0 resected BTC patients: observation, fluoropyrimidine chemoradiation (not recommended for ICC), fluoropyrimidine-based or gemcitabine (GEM)-based chemotherapy and clinical trial.^[10]^ As adjuvant therapy remains controversial in BTC, our retrospective study aim was to evaluate the efficacy of chemotherapy in terms of DFS and OS compared to observation alone in patients with BTC after radical resection and preliminarily discussed the efficacy of different chemotherapy regimens.

## 2 Patients and methods

The present study had been approved by the ethics committee of the Zhejiang Cancer Hospital. Patients with BTC were diagnosed histologically and were classified according to the 2009 American Joint Committee on Cancer (AJCC) staging system.^[11]^ BTC was defined as tumors of the gallbladder and the intrahepatic, perihilar, distal bile ducts, and the ampulla of vater. Based on the hospital database, we retrospectively reviewed 769 patients with BTC from January 2008 to November 2016 at our hospital and finally included 80 patients. The patients enrolled in this study satisfied the following criterion:

(i) histopathologically confirmed adenocarcinoma of BTC;
(ii) R0 resection;
(iii) age 30 to 80 years;
(iv) no previous treatment with chemotherapy or radiotherapy;
(v) an Eastern Cooperative Oncology Group (ECOG) performance status (PS) score of 0 or 1.

Among them, 40 patients had received chemotherapy ≥2 cycles, chemotherapy regimens including GEM, GEM with cisplatin or oxaliplatin or S-1, S-1, capecitabine, and other chemotherapy regimens. In addition to oral chemotherapy S-1 and capecitabine, other chemotherapeutic drugs were administered by intravenous injection. The other 40 patients were chosen 1:1 matched to patients in the former group by gender, age, tumor stage, and ECOG PS score. They received surgery, but no treatment of adjuvant chemotherapy. All cases were followed up by telephone, including recurrence/metastasis events and death events relevant to BTC. Chemotherapy-related toxicity was assessed based on the National Cancer Institute Common Toxicity Criteria scale, version 4.

## 3 Statistics

The primary outcome measure was disease-free survival (DFS), which was defined as time until local or distant disease relapse after treatment. OS was defined as time from diagnosis until death from any cause. All patients were followed until September 2017. Patients who failed to undergo follow-up procedures were censored on the last day when they were confirmed to be alive. Data were analyzed using the SPSS software version 22.0. Upon ensuring the accuracy of data entry and statistical assumptions, descriptive statistics were performed to describe the sample characteristics. Kaplan-Meier analyses were performed to compare the differences of DFS and OS between the 2 groups, and differences in survival curves were compared by log-rank (Mantel-Cox) test. Variables to be analyzed meet the application conditions of Cox proportional hazards model (Supplementary Table 1, 2). Multivariate analysis using the Cox proportional hazards model with entering selection method was performed to adjust for potential confounding factors. Statistical significance was accepted at the level of *P* <.05.

## 4 Results

### 4.1 Patient characteristics

Forty patients received adjuvant chemotherapy and 40 patients were observed. There were no significant differences found in age, gender, PS, tumor location, histologic differentiation, T factor, N factor, stage between 2 groups. Patient demographic and tumor characteristics in the adjuvant chemotherapy and observation groups are summarized in Table 1. Most tumors were located in the common bile duct, 23/40 (57.5%) in the adjuvant chemotherapy group and 18/40 (45.0%) in the observation group.

**Table 1:**
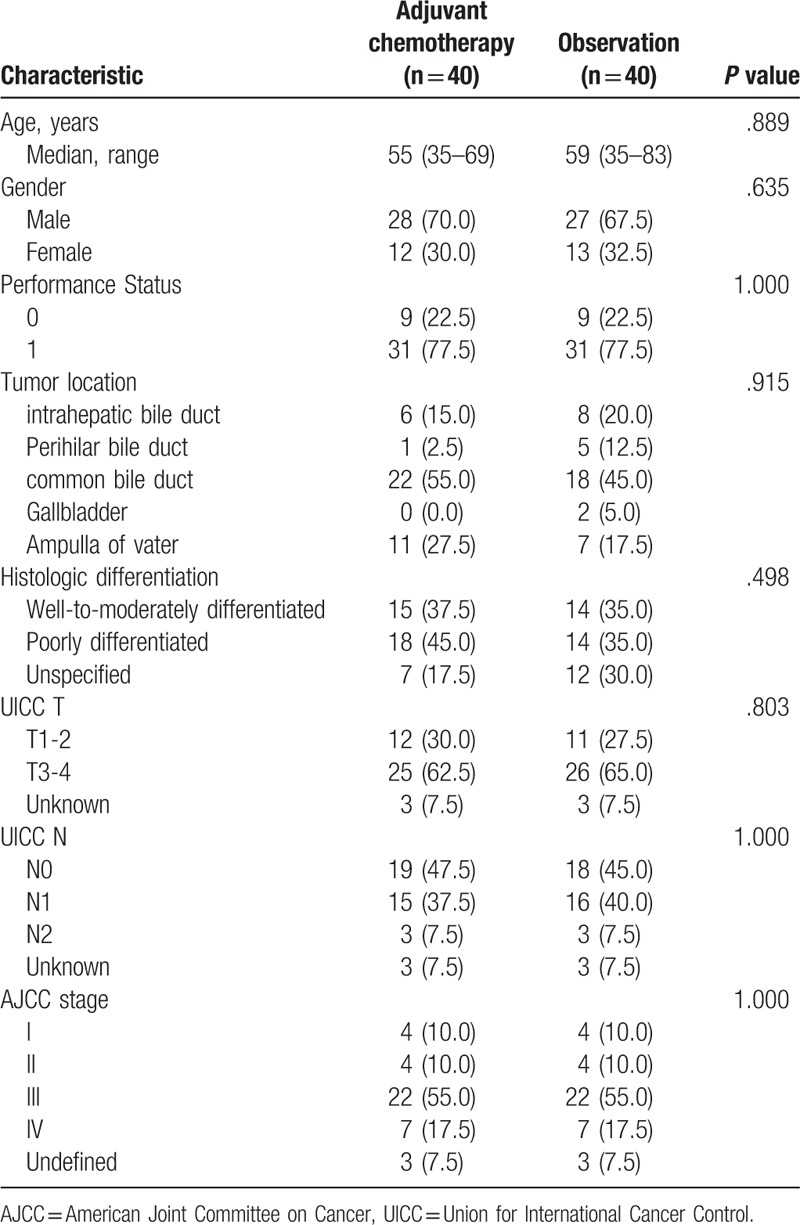
Baseline characteristics of patients (N = 80).

In the adjuvant chemotherapy group, GEM with cisplatin was most commonly used (13/40, 32.5%), followed by S-1 (6/40, 15.0%), GEM with oxaliplatin (5/40, 12.5%), GEM (5/40, 12.5%), GEM with S-1 (4/40, 10.0%), capecitabine (3/40, 7.5%), and other chemotherapy regimens (4/40, 10.0%).

### 4.2 Survival analysis

**Figure 1:**
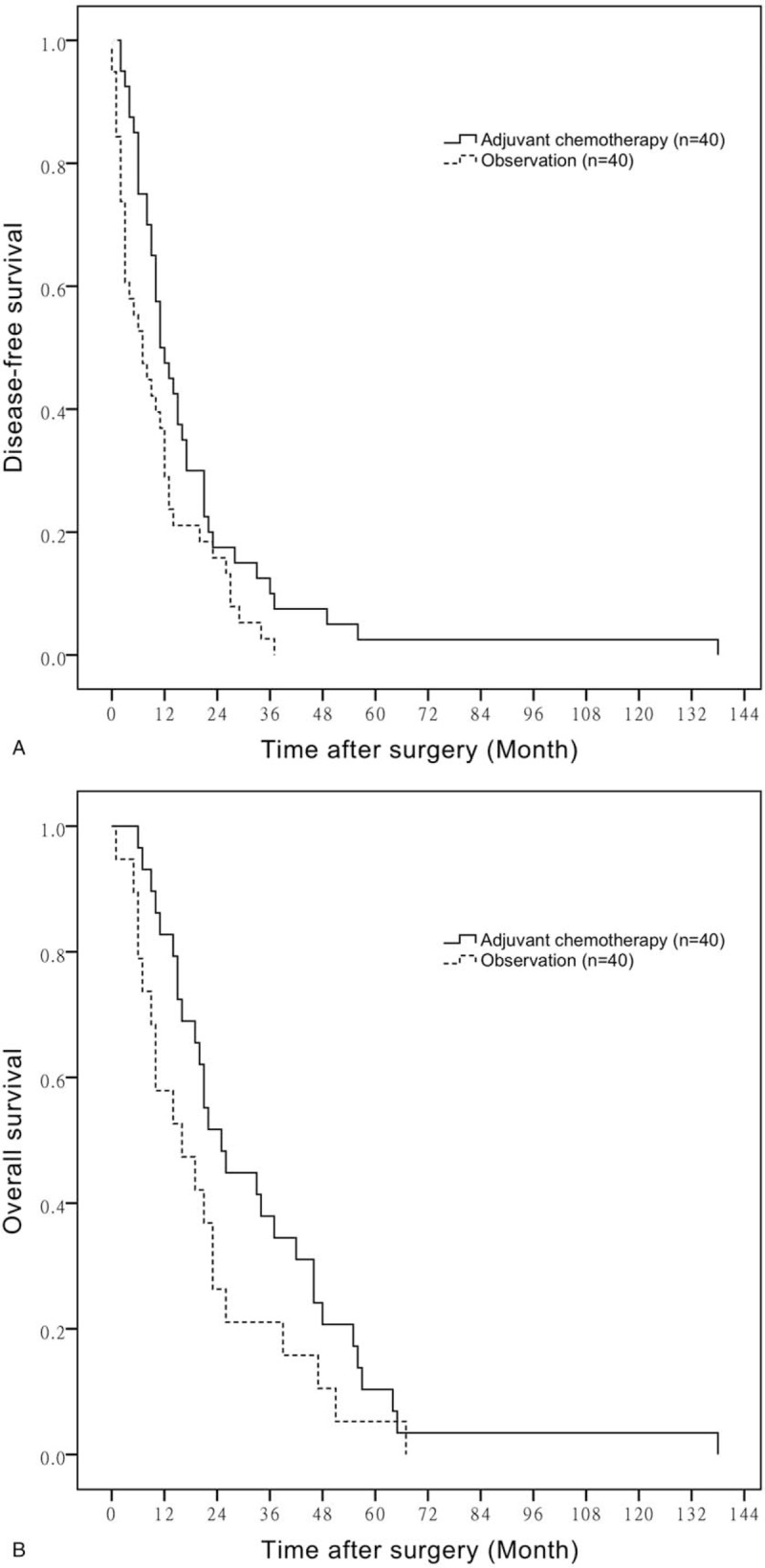
A. DFS of adjuvant chemotherapy compared with observation. B. OS of adjuvant chemotherapy compared with observation. DFS = disease-free survival, OS = overall survival.

During the follow-up period which had a median length of 49.5 months, 11 patients in the adjuvant chemotherapy group and 20 patients in the observation group died of disease-related causes. The mean DFS time of BTC patients with adjuvant chemotherapy and observation was 18.63 ± 3.63 months versus 10.36 ± 1.67 months, respectively (*P* = 0.029). The probabilities of 1-, 2-, and 3-year DFS in the adjuvant chemotherapy group were 50.0, 17.5, and 12.5%, while those in the observation group were 36.8, 15.7, and 2.6%, respectively (Fig. 1A). The mean OS time of BTC patients with adjuvant chemotherapy and observation is 33.72 ± 5.02 versus 21.05 ± 4.12 months, respectively (*P* = .114). The probabilities of 1-, 2-, and 3-year survival in the adjuvant chemotherapy group were 82.8, 51.7, and 37.9%, while those in the observation group were 57.9, 26.3, and 21.1%, respectively (Fig. 1B). On multivariate analysis, 7 variables for predicting survival were incorporated: adjuvant chemotherapy, sex, age, T factor, N factor, tumor stage, and PS. Of these variables, adjuvant chemotherapy and N factor were found to be significant factors for DFS, and sex, age, T factor were found to be significant factors for OS (Table 2).

**Table 2:**
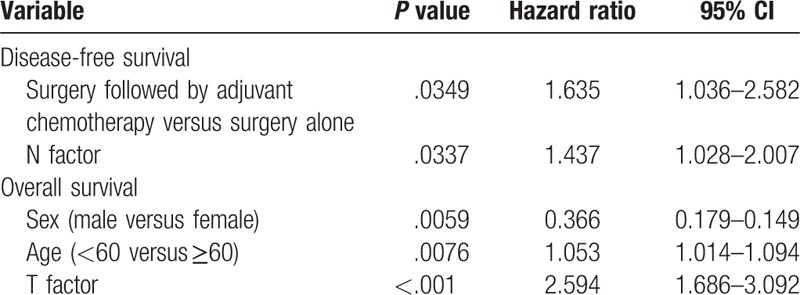
Multivariate analysis of clinicopathologic factors associated with DFS and OS.

### 4.3 Adverse events

Throughout the entire treatment, hematological toxic events were commonly observed in the chemotherapy group (Table 3). Grade 3 to 4 leucopenia, granulopenia, anemia, and thrombocytopenia were observed in 8 (8/40,20%), 16 (16/40,40%), 5 (13/40,13%), and 6 (6/40,15%) of patients, respectively. 8 (8/40,20%) patients underwent Grade III to IV hepatic function damage according to CTCAE v4.0. There were no treatment-related deaths, and all the toxic effects were manageable.

**Table 3:**
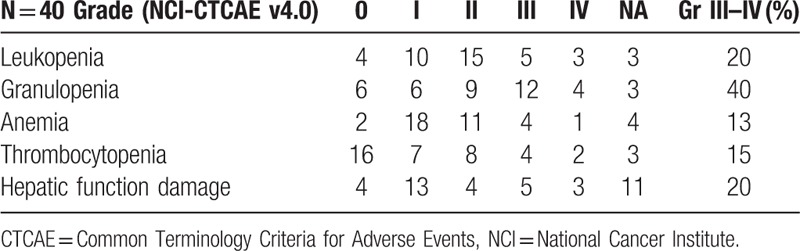
Chemotherapy toxicities.

### 4.4 Chemotherapy regimens comparison (Table 4)

**Figure 2:**
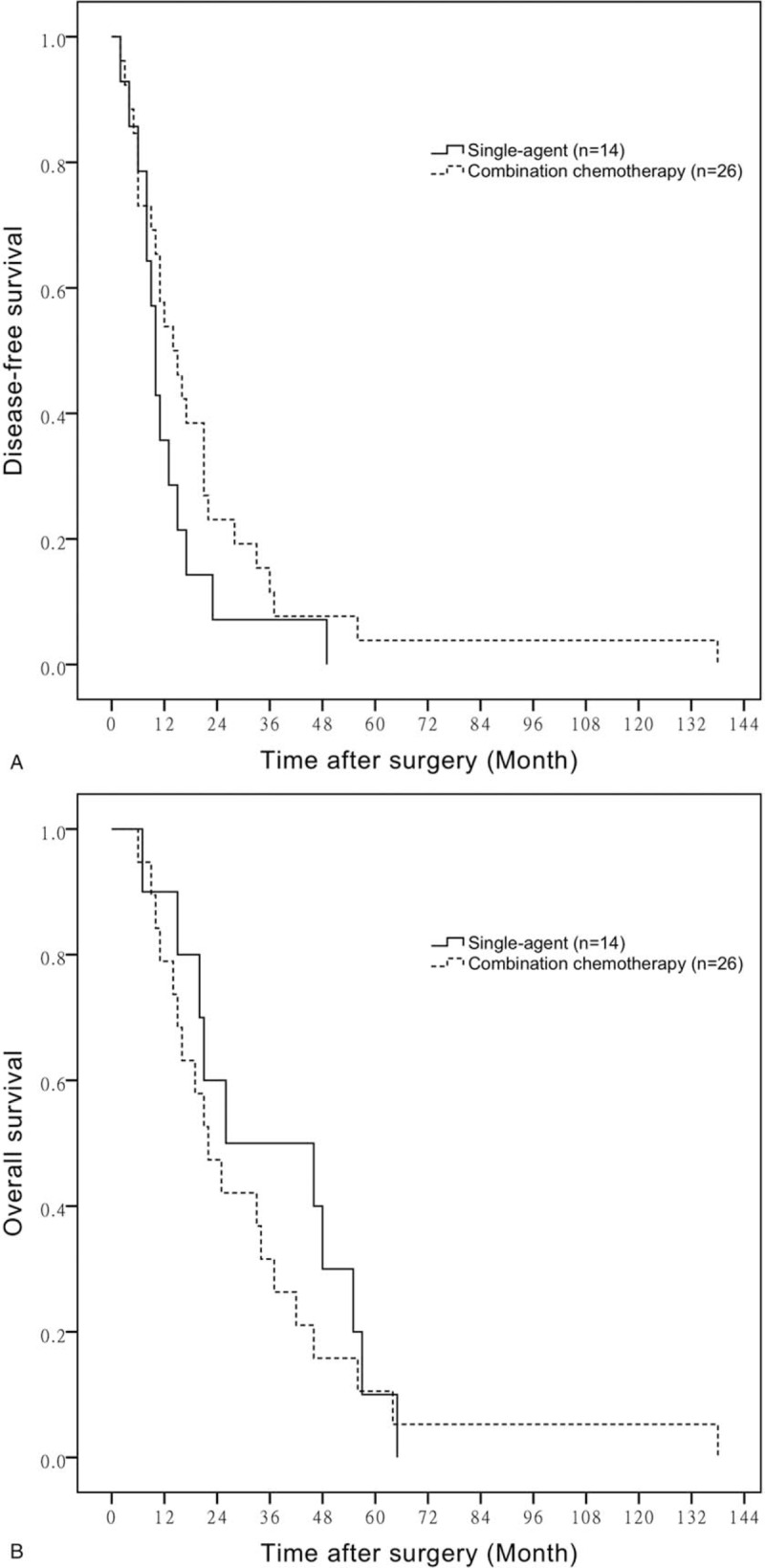
A. Comparison of DFS in patients with single-agent chemotherapy or observation. B. Comparison of OS in patients with single-agent chemotherapy or observation. DFS = disease-free survival, OS = overall survival.

We define BTC patients who received more than one agent as combination chemotherapy group, while those who had GEM or S-1 or capecitabine only as single-agent group. We then compared patients’ outcome in combination chemotherapy group with single-agent group. There were no significant differences observed in age, gender, N factor and chemotherapeutic cycles between the 2 groups, but patients in combination chemotherapy group have poorer PS, T factor and higher stage than single-agent group. The mean DFS time of BTC patients with combination chemotherapy and single-agent was 21.54 ± 5.29 months versus 13.21 ± 3.11 months, respectively (*P* = .185) (Fig. 2A). The mean OS time of BTC patients in 2 groups is 36.00 ± 6.46 versus 32.53 ± 7.00 months, respectively (*P* = .494) (Fig. 2B).

**Table 4:**
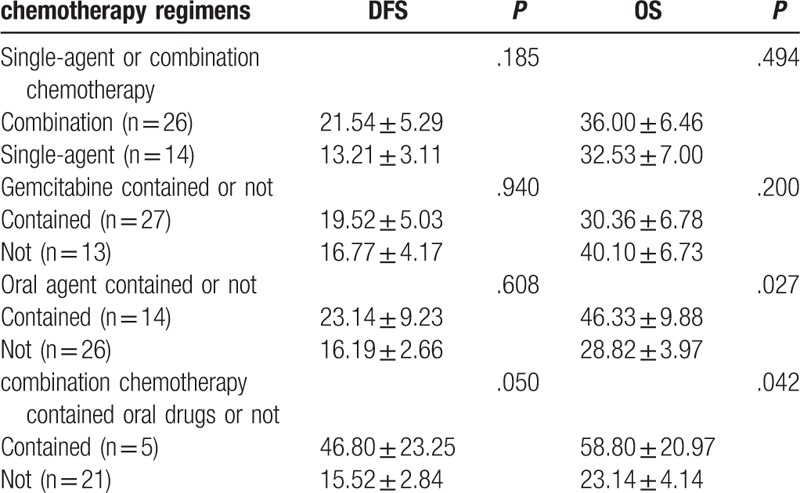
Chemotherapy regimens comparison.

**Figure 3:**
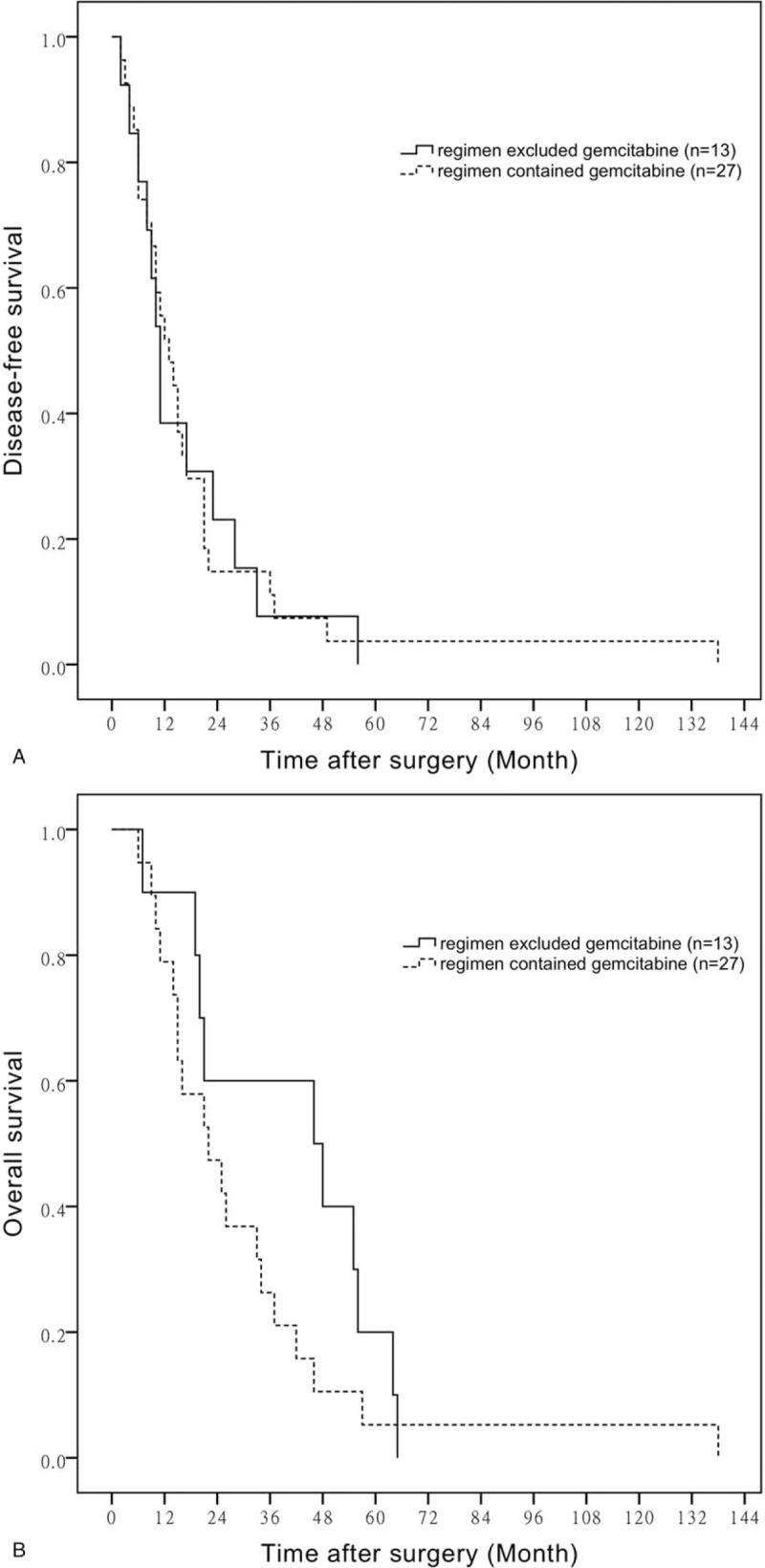
A. Comparison of DFS in patients with regimens contained gemcitabine or not. B. Comparison of OS in patients with regimens contained gemcitabine or not. DFS = disease-free survival, OS = overall survival.

BTC patients whose regimens had contained GEM presented a DFS time 19.52 ± 5.03 months versus those who had not were 16.77 ± 4.17 months (P = 0.940) (Fig. 3A). The mean OS time of BTC patients in GEM contained group is 30.36 ± 6.78 months versus regimens without GEM 40.10 ± 6.73 months (*P* = .200) (Fig. 3B).

**Figure 4:**
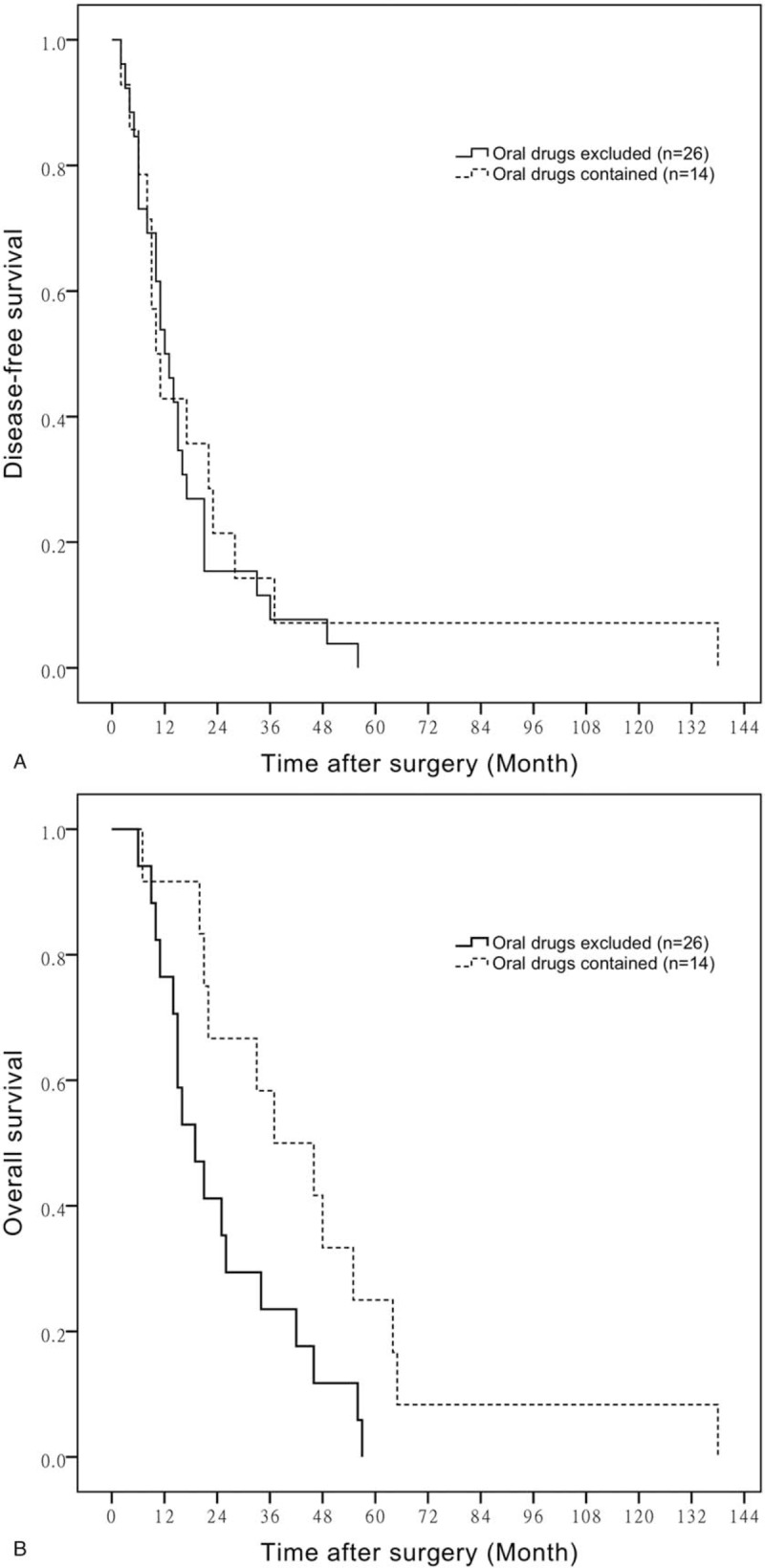
A. DFS of oral drugs contained chemotherapy compared with chemotherapy excluded oral drugs. B. OS of oral drugs contained chemotherapy compared with chemotherapy excluded oral drugs. DFS = disease-free survival, OS = overall survival.

Currently, oral chemotherapy drugs used in BTC patients are S-1 and capecitabine. We define patients who received S-1 or capecitabine in their regimens as oral drugs contained group, while those who not as oral drugs excluded group. The mean DFS time of BTC patients who had taken oral drugs and those had not is 23.14 ± 9.23 months versus 16.19 ± 2.66 months, respectively (*P* = .608) (Fig. 4A). However, the mean OS time of BTC patients is 46.33 ± 9.88 versus 28.82 ± 3.97 months in the 2 groups (*P* = .027) (Fig. 4B).

**Figure 5:**
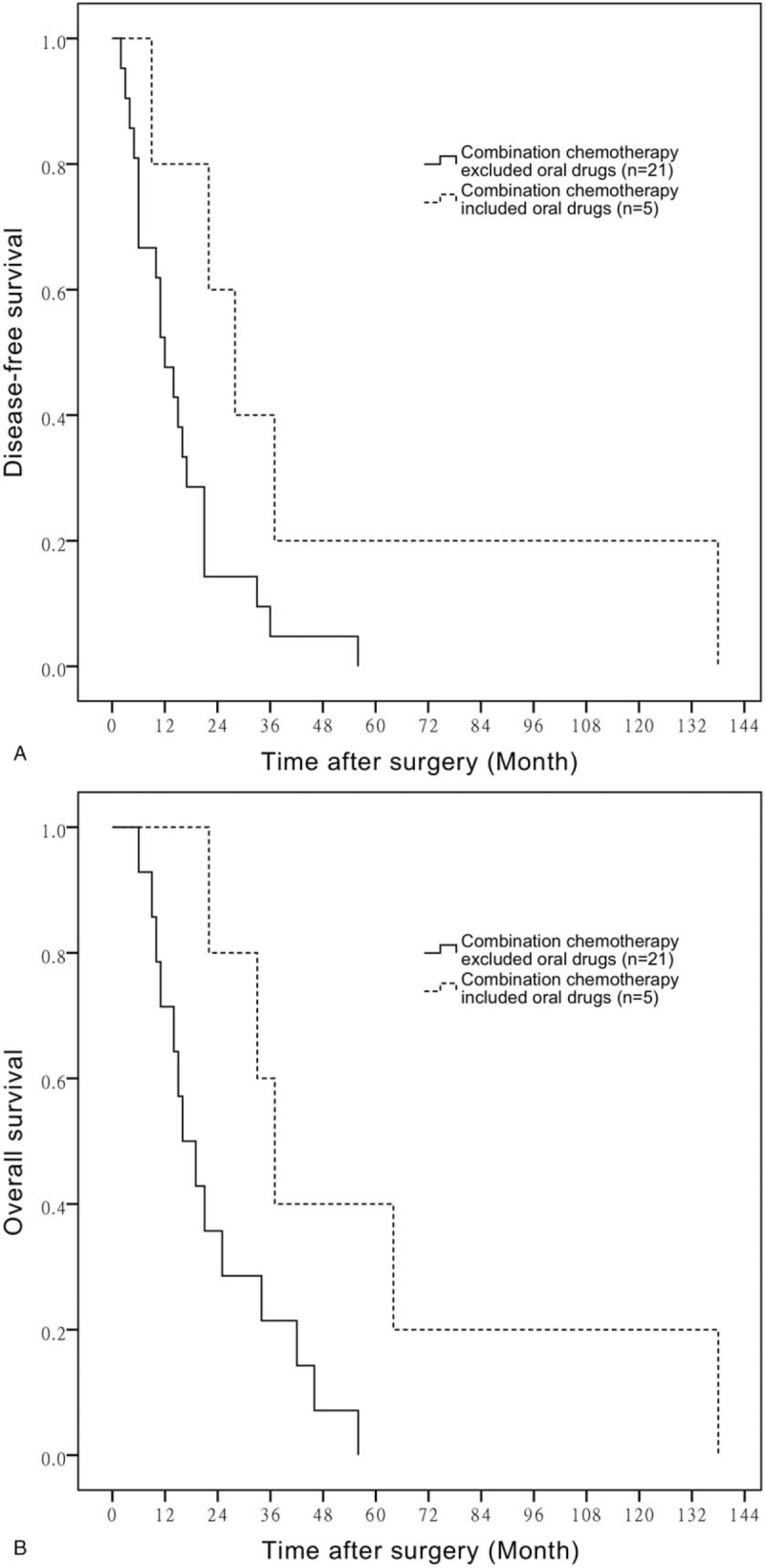
A. Comparison of DFS in patients with combination chemotherapy contained oral drugs or not. B. Comparison of OS in patients with combination chemotherapy contained oral drugs or not. DFS = disease-free survival, OS = overall survival.

Additionally, the mean DFS time of BTC patients who had received combination chemotherapy contained oral agents or not is 46.80 ± 23.25 months versus 15.52 ± 2.84 months, respectively (*P* = .050) (Fig. 5A). The mean OS time of BTC patients who were given oral agents as a part of combination chemotherapy and not is 58.80 ± 20.97 versus 23.14 ± 4.14 months, respectively (*P* = .042) (Fig. 5B).

## 5 Discussion

Some evidence shows adjuvant chemotherapy should be effective in R0 resected BTC patients. However, the standard regimen for adjuvant chemotherapy is not determined currently. In locally advanced or metastatic BTC cases, chemotherapy contained GEM is considered as standard regimen. Kenya Yamanaka et al performed a historical cohort study that involved 198 patients who underwent R0 surgical resection, 40 of them were received gemcitabine after surgery. The author came to the conclusion that a significant increase in survival due to the administration of adjuvant GEM (GEM) chemotherapy according to HR = 0.47 [95% confidence interval (CI) 0.23–0.95; P = 0.04].^[12]^ In a randomized, multidisciplinary, multinational phase III trial ACTICCA-1, 2 separate cohorts (280 patients in the CCA cohort and 160 patients in the GBCA cohort) both showed potential advantage of the combination of GEM and cisplatin used as postoperative treatment.^[13]^ In addition to GEM, oral fluoropyrimidines are considered to be the most promising agents with mild toxicity for the treatment of advanced BTC.^[14]^ Young Saing Kim et al retrospectively analyzed the clinical data of 153 BTC patients who underwent curative-intent R0 resection, 5-year OS rates (48.4% vs 39.6%, *P* = .439) and 3-year recurrence-free survival (RFS) rates (49.1% vs 39.5%, *P* = .299) between patients who received fluoropyrimidine-based adjuvant chemotherapy or observation both revealed no advantage of adjuvant chemotherapy. However, for patients with stages II and III BTC, chemotherapy significantly improved 5-year OS rate (52.4% vs 35.6%, *P* = .002) and 3-year RFS rate (55.5% vs 39.1%, *P* = .021) compared with observation.^[15]^

This analysis including 80 BTC patients with R0 resection from 2008 to 2016 demonstrates that adjuvant chemotherapy contributes to DFS, but is unsatisfactory for improving OS. The role of adjuvant chemotherapy to improved DFS was confirmed by multivariate analysis. Our case–control study adjusted confounders as patients were 1:1 matched by clinical characteristics, which was conductive to draw a convinced conclusion. Besides, the highlight of this study is that the effect of different chemotherapy regimens was discussed to provide clues for the most effective chemotherapy choice. E. Una Cidon proposed in his review that it could be ponderable to probe into that single-agent versus doublet chemotherapy which shows more benefits for survival.^[16]^ Our first subgroup was an analysis of the single-agent versus the combination chemotherapy, although patient in combination chemotherapy group has poorer PS and more advanced tumor stage, they still tend to have a longer disease-free survival, which indicated that combination chemotherapy can better restrain tumor recurrence. As GEM is commonly used in BTC patients, we attempted to see if the chemotherapy regimen contained GEM would affect patients’ survival. A comparison of regimens contained GEM or not in our study indicated that GEM-based chemotherapy did not show superiority than other regimens. There was a significant difference of OS time between patients who had taken oral drugs and those not, the difference was not seen in DFS time. We reviewed our clinical data and found that 75.0% patients in oral drug group received palliative chemotherapy while 41.2% patients without oral drug did when tumor recurred. Patients who received oral chemotherapy as adjuvant and subsequently also received systemic therapy on recurrence, likely improved their OS. The prolonged OS time may be caused by palliative chemotherapy. Result from last subgroup indicated that patient who had taken combination chemotherapy within oral agents presented longer DFS and OS time than those had combination chemotherapy without oral agents. It has been revealed that capecitabine has promising therapeutic effect, but single agent may not be enough. While a randomized controlled phase III trial with a larger number of patients is required to confirm the efficacy of combination chemotherapy, the use of oral agents is an active therapeutic option for improving surgical outcomes of patients with BTC. We are interested if patients can profit more from capecitabine combined with other drugs like GEM.

The patients included in our study tended to have a shorter survival time than anterior studies, and that may be caused by the higher tumor stage of our patients, more than half of whom were defined as stage III. Besides, there are some limitations existing in our study including the small number of patients and the participants had different types of BTCs, as well as farraginous regimes. And the study was non-random. In addition, the subgroups were not organized in a strict way, and unselected variables that might affect the survival were not adjusted, which rendered the results only provide a possibility of therapeutic strategy rather than a strong evidence.

## Author contributions

**Data curation:** Luxi Yin, Jieer Ying, Qi Xu, Jingjing Li.

**Formal analysis:** Luxi Yin.

**Investigation:** Luxi Yin, Qi Xu, Jingjing Li, Qing Wei.

**Methodology:** Luxi Yin, Jieer Ying, Qi Xu.

**Software:** Luxi Yin.

**Writing – original draft:** Luxi Yin.

**Conceptualization:** Jieer Ying.

**Project administration:** Jieer Ying.

**Resources:** Jieer Ying, Qi Xu, Jingjing Li, Qing Wei.

**Supervision:** Jieer Ying.

**Validation:** Jieer Ying, Qing Wei.

**Visualization:** Jieer Ying, Qi Xu, Jingjing Li.

**Writing – review & editing:** Jieer Ying.

## Supplementary Material

Supplemental Digital Content

## References

[1] Eric IM Nelson SY Immunotherapeutic approaches in biliary tract carcinoma: current status and emerging strategies. World J Gastrointest Oncol 2015;7:338–46. 26600933 10.4251/wjgo.v7.i11.338 PMC4644856

[2] Dingle BH Rumble RB Brouwers MC The role of gemcitabine in the treatment of cholangiocarcinoma and gallbladder cancer: a systematic review. Can J Gastroenterol 2005;19:711–6. 16341310 10.1155/2005/565479

[3] Miyazaki Y Kokudo T Amikura K Survival of surgery for recurrent biliary tract cancer: a single-center experience and systematic review of literature. Jpn J Clin Oncol 2017;47:206–12. 27940488 10.1093/jjco/hyw182

[4] Andrew XZ Theodore SH Aram FH Current management of gallbladder carcinoma. Oncologist 2010;15:168–81. 20147507 10.1634/theoncologist.2009-0302 PMC3227945

[5] Marinelli I Guido A Fuccio L Clinical target volume in biliary carcinoma: a systematic review of pathological studies. Anticancer Res 2017;37:955–62. 28314252 10.21873/anticanres.11404

[6] Vedat G Cholangiocarcinoma: new insights. Asian Pac J Cancer Prev 2017;18:1469–73. 28669153 10.22034/APJCP.2017.18.6.1469 PMC6373807

[7] Kalyan CM Fatima H Hammad S Adjuvant therapy for resected gallbladder cancer: analysis of the national cancer data base. J Natl Cancer Inst 2016;109:pii: djw202. 10.1093/jnci/djw202 27707843

[8] Se IG Young SK In GH Is There a role for adjuvant therapy in r0 resected gallbladder cancer?: a propensity score-matched analysis. Cancer Res Treat 2016;48:1274–85. 26875193 10.4143/crt.2015.502 PMC5080804

[9] Timur M C Kristian E Ahmedin J Limited use of adjuvant therapy in patients with resected gallbladder cancer despite a strong association with survival. J Natl Cancer Inst 2017;109: doi: 10.1093/jnci/djw324. 10.1093/jnci/djw324 28376178

[10] National Comprehensive Cancer Network. NCCN Guidelines for Treatment of Cancer by Site: Hepatobiliary Cancers (version 1.2018). Available at: https://www.nccn.org/professionals/physician_gls/pdf/hepatobiliary.pdf Accessed February 14, 2018.

[11] Edge SB Byrd DR Compton CC The American Joint Committee on Cancer: the 7th edition of the AJCC cancer staging manual and the future of TNM. Ann Surg Oncol 2010;17:1471–4. 20180029 10.1245/s10434-010-0985-4

[12] Yamanaka K Hatano E Kanai M A single-center analysis of the survival benefits of adjuvant gemcitabine chemotherapy for biliary tract cancer. Int J Clin Oncol 2014;19:485–9. 23765238 10.1007/s10147-013-0578-x

[13] Alexander S Dirk A John B Adjuvant chemotherapy with gemcitabine and cisplatin compared to observation after curative intent resection of cholangiocarcinoma and muscle invasive gallbladder carcinoma (ACTICCA-1 trial) - a randomized, multidisciplinary, multinational phase III trial. BMC Cancer 2015;15:564. 26228433 10.1186/s12885-015-1498-0 PMC4520064

[14] Takashi S Hiroyuki I Yousuke N Current status of chemotherapy for the treatment of advanced biliary tract cancer. Korean J Intern Med 2013;28:515–24. 24009445 10.3904/kjim.2013.28.5.515 PMC3759755

[15] Young SK Chi YJ Haa NS The efficacy of fluoropyrimidine-based adjuvant chemotherapy on biliary tract cancer after R0 resection. Chin J Cancer 2017;36:9. 28086990 10.1186/s40880-017-0182-y PMC5237216

[16] E. Una C Resectable cholangiocarcinoma: reviewing the role of adjuvant strategies. Clin Med Insights Oncol 2016;10:43–8. 27199577 10.4137/CMO.S32821 PMC4869598

