# Supplementary material for: The efficiency and regimen choice of adjuvant chemotherapy in biliary tract cancer: A STROBE-compliant retrospective cohort study
Source: Medicine (Baltimore). 2018 Dec 14;97(50):e13570. doi: 10.1097/MD.0000000000013570 (PMC6320011; doi:10.1097/MD.0000000000013570)

Supplementary Table 1.*Variables associated with DFS and proportional hazards assumption*


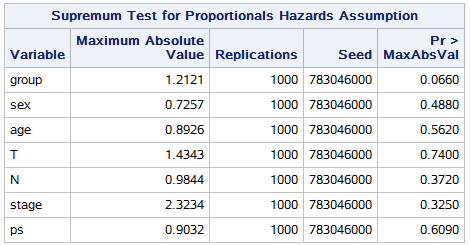


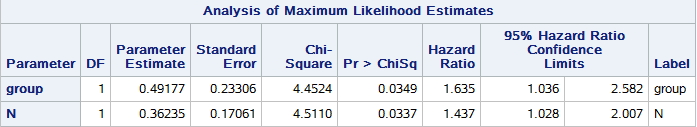


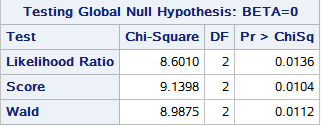


Supplementary Table 2.*Variables associated with OS and proportional hazards assumption*


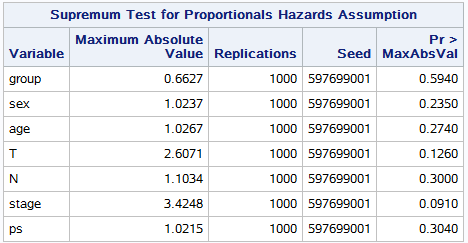


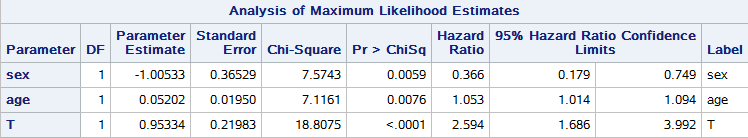


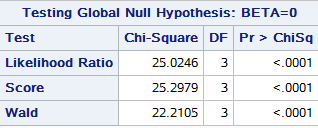

Supplement: Supplemental Digital Content [file medi-97-e13570-s001.docx]
